# Intravenous Uses of Formalin in the Treatment of Septicemia

**Published:** 1903-04

**Authors:** 


### INTRAVENOUS USES OF FORMALIN IN THE TREATMENT OF SEPTICEMIA.

The case of puerperal sepsis treated by this methord by Dr. Barrow at Bellevue hospital was given much newspaper notoriety and was hailed as a specific But more temporate judgement flanked by physological experiment has not established it is such a light. Some successes have been noted and some failures, the latter preponderating—Physiological experiments upon animals do not however prove the method a failure for the reaction in man may not be the same as in small animals as was known by the laboratory success of antistreptoccius serum and its clinical failure. The collected evidence in the matter of the formalin method appears to place it in the category of therapeutic measures of doubtful cilility but which may be used on occasion *faute de mieux.*

